# Neural Dynamics during Anoxia and the “Wave of Death”

**DOI:** 10.1371/journal.pone.0022127

**Published:** 2011-07-13

**Authors:** Bas-Jan Zandt, Bennie ten Haken, J. Gert van Dijk, Michel J. A. M. van Putten

**Affiliations:** 1 Neuroimaging at MIRA-Institute for Biomedical Technology and Technical Medicine, University of Twente, Enschede, The Netherlands; 2 Department of Neurology, Leiden University Medical Center, Leiden, The Netherlands; 3 Department of Clinical Neurophysiology, Medisch Spectrum Twente, Enschede, The Netherlands; 4 Clinical Neurophysiology at MIRA-Institute for Biomedical Technology and Technical Medicine, University of Twente, Enschede, The Netherlands; Hôpital Robert Debré, France

## Abstract

Recent experiments in rats have shown the occurrence of a high amplitude slow brain wave in the EEG approximately 1 minute after decapitation, with a duration of 5–15 s (van Rijn et al, PLoS One 6, e16514, 2011) that was presumed to signify the death of brain neurons. We present a computational model of a single neuron and its intra- and extracellular ion concentrations, which shows the physiological mechanism for this observation. The wave is caused by membrane potential oscillations, that occur after the cessation of activity of the sodium-potassium pumps has lead to an excess of extracellular potassium. These oscillations can be described by the Hodgkin-Huxley equations for the sodium and potassium channels, and result in a sudden change in mean membrane voltage. In combination with a high-pass filter, this sudden depolarization leads to a wave in the EEG. We discuss that this process is not necessarily irreversible.

## Introduction

Oxygen and glucose deprivation has almost immediate effects on brain function, typically causing symptoms in approximately 5–7 seconds. This dysfunction is also reflected in the electroencephalogram (EEG), generally consisting of an increase in slow wave activity and finally in the cessation of activity. These phenomena are a direct consequence of synaptic failure of pyramidal cells [1], reflecting the high metabolic demand of synaptic transmission [2].

**Figure 1:**
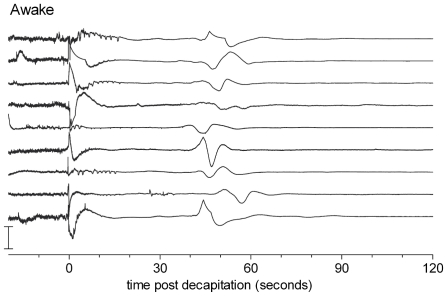
EEGs recorded in 9 animals after decapitation. Note the large slow wave around 50 s after decapitation. Similar experiments were performed in an anesthetized group of animals, where the wave appeared at a slightly later instant, approximately 80 s. The changes in amplitude at t = 0 are movement artifacts due to the decapitation. Figure from [3].

Recent findings in rats, decapitated to study whether this is a humane method of euthanasia in awake animals, indeed showed disappearance of the EEG signal after approximately 15–20 s. After half a minute of electrocerebral silence, however, a slow wave with a duration of approximately 5–15 seconds appeared (Figure 1). It was suggested that this wave might reflect the synchronous death of brain neurons [3] and was therefore named the “Wave of Death”.

Similar experiments were performed by Swaab and Boer in 1972 [4]. The EEG survival time was of the same order as the observations of van Rijn et al [3]: after approximately 7 s the EEG flattened to become iso-electric after 20 s. Recordings did not last longer than that, however, which may explain why the “Wave of Death” was not detected in these experiments.

Van Rijn et al. [3] speculated that the wave might be due to a simultaneous and massive loss of resting membrane potential, caused by the oxygen-glucose deprivation (OGD) following decapitation. Indeed, plenty of (experimental) literature exists showing that hypoxia causes membrane depolarization. Siemkowicz and Hansen [5], for instance, induced complete cerebral ischemia in rats for ten minutes. During and after this period they recorded an EEG and measured the extracellular potential and extracellular ion concentrations. A rapid deflection of the extracellular potential occurred typically 1–2 minutes after the onset of ischemia, accompanied by a sudden rise in extracellular potassium. Unfortunately, EEG activity during the ischemic episode was not described and it is unknown whether a similar wave in the EEG occurred here. Another example is the work of Dzhala et al., who perfused rat brains in vivo with an anoxic-aglycemic solution and measured the transmembrane potential of a pyramidal cell. Approximately eight minutes after the onset of the induced ischemia, they observed a rapid depolarization of the cell membrane [6]. Depolarization is also observed in computational models. For example, Kager et al. modeled neuronal dynamics and ion concentrations and show that an increased concentration of potassium in the neuronal environment can cause fast membrane depolarizations. Depolarization also takes place in their simulations when the ion pump rates are lowered and a neuron is stimulated by injecting current for a few 100 ms [7], [8].

In this communication we present a minimal biophysical, single-cell model. Using Hodgkin-Huxley dynamics to describe the voltage-dependent ion channel dynamics, including oxygen/glucose dependent ion pumps, we show that severe oxygen-glucose deprivation results in a sudden depolarization of the membrane voltage. Subsequent modeling of the EEG results in a macroscopic wave, as observed by van Rijn et al. [3]. Finally we discuss that this wave does not reflect irreversible damage and hence not death.

## Methods

### Biophysical model

A biophysically realistic neuron is modeled using Hodgkin-Huxley dynamics of sodium and potassium channels combined with leak currents. The model includes the dynamics of the extra- and intracellular ion concentrations, which change significantly when homeostasis cannot be maintained by neurons and glia. Ion pump fluxes are incorporated to model this homeostasis. Our model is based on the equations by Cressman et al [9]–[11], who studied the effects of the extracellular ion concentrations in the generation of epileptic seizures.

The model consists of an intracellular and an extracellular compartment separated by a semi-permeable cell membrane. This membrane contains a fast transient sodium channel, a delayed rectifier potassium channel and a leak for sodium, potassium and chlorine. The dynamics of the membrane voltage, 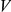, are described with the Hodgkin-Huxley equations:
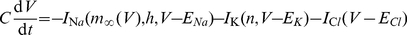
(1)with 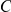 the membrane capacitance and 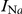, 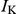, 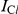 the total sodium, potassium and chloride currents. The Nernst potential for each ion species is indicated with 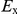 and given by 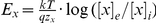, with 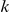 the Boltzmann constant, 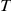 the absolute temperature, 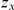 the valency of the ion, 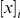 and 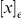 the intra- and extracellular concentrations and x  =  Na, K, Cl. The fraction of activated sodium channels, 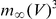 is due to its fast dynamics assumed to depend instantaneously on the membrane voltage. 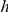 is the fraction of inactivated sodium channels and is a variable in our model. 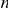 is the fraction of activated potassium channels and is also a variable. The calcium gated current from the Cressman model is not implemented, because it does not qualitatively alter the behavior of interest here. We write for the total sodium, potassium and chloride currents
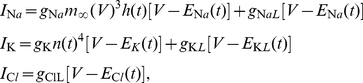
(2)respectively. The maximum ion conductances for the gated currents are denoted with 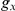 and for the leak currents with 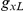.

The gating variables 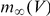, 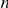 and 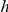 are modeled as [11]:
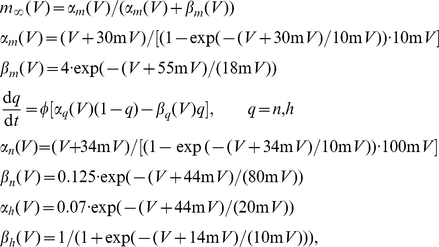
(3)where 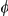 is the time constant of the channels. When the ion concentrations, on which the Nernst potentials depend, are assumed to be constant, equation sets 1 to 3 can be used to model the dynamical behavior of a single neuron.

In order to calculate changes in ion concentrations in the model, equations are added that integrate the ion fluxes into and out of the two compartments. During physiological conditions, the concentrations are given by [11]:
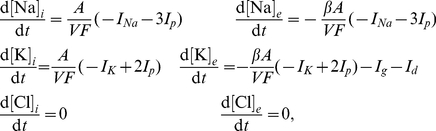
(4)with A and V respectively the surface area and volume of the cell, F the Faraday constant and 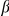 the ratio of the intra- and extracellular volumes. 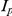 denotes a sodium-potassium pump current (in 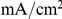) which depends sigmoidally on the intracellular sodium concentration and the extracellular potassium concentration. The total amount of sodium is preserved in this model, but the extracellular potassium can be buffered by glial cells (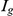) and can diffuse from and into the blood (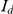). Furthermore, the chlorine concentrations are assumed to remain constant under normal conditions, without specifying the mechanism for this. The approximation that the efflux of potassium equals the influx of sodium made by Cressman et al. in order to reduce the number of variables is not made here.

The pump, glial and diffusion currents are modeled as [11]:
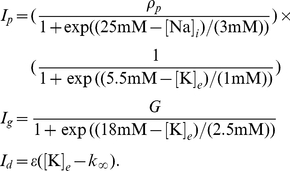
(5)


Here 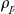 scales the pump rate, 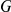 the glial buffering rate, 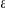 is the time constant of diffusion and 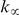 the concentration of potassium in the blood. Note that 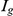 and 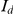 do not have the dimension of current, but that of rate of change of concentration (mM/s).

### Numerical implementation

Equation sets 1 to 5 completely describe our model. The resting state of this system is calculated, with the parameters shown in table 1. The equations were solved with a solver for stiff ordinary differential equations (ode23 routine, Matlab, the Mathworks). The simulation code is available from ModelDB [12], accession number 139266. Table 2 shows the results of this calculation, which are used as starting point for the simulation of oxygen and glucose deprivation. It was verified that the model behaves as expected under normal circumstances: in rest the membrane potential and the sodium and potassium concentrations are in the physiological range. Furthermore the neuron responds with a single action potential when a short current pulse is applied and spikes periodically when a current of 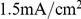 or more is injected.

**Table 1:** Overview of the parameters used in the simulations.

variable	value	units	description
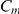	1.0	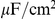	specific membrane capacitance
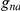	100	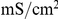	sodium channel conductance
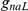	0.0175	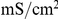	sodium leak conductance
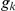	40	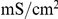	potassium channel conductance
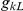	0.05	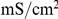	potassium leak conductance
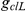	0.05	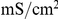	chlorine leak conductance
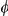	3	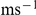	time constant of gating variables
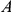 / 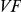	0.044	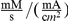	conversion factor current to concentration
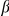	2.0		ratio intra-/extracellular volume
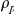	28.1	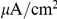	NaK-Pump rate
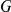	66	mM/s	glial buffering rate of 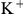
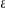	1.3	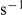	diffusion rate
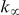	4.0	mM	concentration 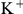 in blood
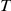	310	K	absolute temperature

**Table 2:** Overview of the variables in the steady state.

variable	steady state value	units
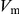	-68	mV
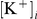	139	mmol
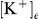	3.8	mmol
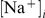	20	mmol
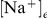	144	mmol
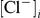	6.0	mmol
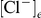	130	mmol

### Simulating oxygen and glucose deprivation

To simulate the anoxia and aglycemia, we set both the pump current and the uptake of 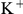 ions by the glial cells to zero as well as diffusion of 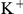 to the blood. Furthermore the chlorine concentrations are no longer assumed to stay constant. This changes the equations for the concentration dynamics, Eqns 4, into:
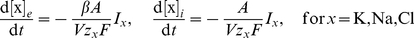
(6)


## Results

**Figure 2:**
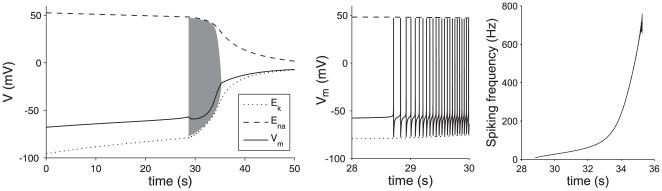
Membrane dynamics during oxygen-glucose deprivation. In the left panel the membrane dynamics are shown that occur after the onset of OGD (solid line). The dashed and dotted lines show the progressive loss of ion gradients. When after a gradual rise the membrane potential reaches the excitation threshold, this subsequently results in spiking of the membrane voltage according to Eqns 1 and 2 (gray region, not resolved). The black line shows the average membrane potential during the spiking (averaged over 300 ms). After approximately 7 seconds of oscillations, the cell comes to rest again, with a resulting 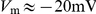. The middle panel shows a close up of the start of spiking activity, the right panel shows the instantaneous firing rate.

In the case of a normally functioning neuronal unit, which maintains homeostasis, the model reaches a steady state with a membrane potential and ionic concentrations in physiological ranges (Table 2). Figure 2 shows the result of our simulation of oxygen and glucose deprivation using this steady state as a starting point. Initially, over the course of half a minute, the membrane voltage rises by approximately 0.7 mV/s. This is due to the efflux of potassium, which causes a rise in 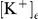 and correspondingly in 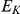. The rise in 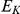 is only partially compensated by the fall of 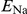, caused by the influx of sodium ions.

At t  =  28.7 s, the resting membrane voltage reaches the excitation threshold, such that the resting state of the cell loses stability and the cell starts to generate action potentials (spikes) with an initial frequency of 10 Hz, increasing to 500 Hz in a 7 s period.

Each spike temporarily opens the potassium channels and transiently increases the efflux of potassium. The resulting increase of the extracellular potassium concentration in turn increases the mean membrane voltage and spiking frequency, forming a positive feedback loop. As a result, the mean membrane potential (Figure 2, left panel) steeply rises from −50 to −20 mV in the last 2 seconds of this oscillation period. During this 2 s period, the amplitude of the action potential spikes decreases to zero, after which the neuron obtains a stable resting state again. In this state, however, the neuron is no longer excitable, due to the so-called depolarization block, i.e. the permanent inactivation of the sodium channels. After the neuron stops spiking, the leak currents cause the difference between the Nernst potentials of sodium and potassium to slowly vanish over the course of a minute. Due to the small chlorine leak the Nernst potentials and membrane voltage eventually reach −20 mV after about ten minutes (not shown).

**Figure 3:**
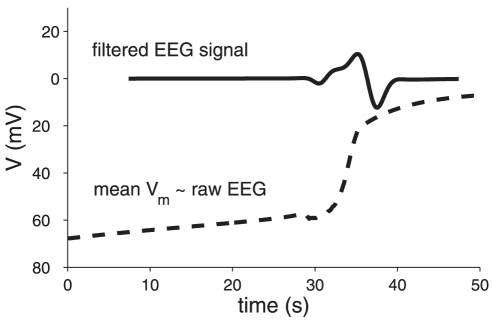
Mean membrane potential and simulated EEG signal. Shown are (dashed line) the simulated membrane potential averaged over 300 ms and in (solid line, a.u.) the signal that results after applying a high-pass filter (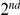 order Butterworth filter, cut-off at 0.1 Hz).

In order to compare the simulated single cell behavior with the EEG observed by van Rijn et al. [3], we proceed as follows. The contribution of a single cell to the (raw) EEG is roughly proportional to its membrane potential [13]. Modeling the EEG realistically usually requires a large scale simulation with many neurons, because the behavior of a cell depends heavily on its interaction with other neurons. The present situation provides an exception, however, because synaptic transmission has stopped and neurons receive no direct input. As a result, their dynamics can be accurately described with a single cell model; the EEG of an ensemble of cells can be calculated by simply summing the contributions of individual neurons. Assuming that many neurons behave approximately the same as the modeled neuron, but with some small shift in time, the resulting raw EEG is proportional to the mean membrane potential (Figure 3, dashed line). For simplicity, a flat distribution of 300 ms wide was chosen, but varying the shape and width of this distribution hardly changes the resulting EEG. High-pass filtering the resulting potential with a cut-off at 0.1 Hz replicates the filter characteristics of the filter used by van Rijn et al. [3]. This results in the solid curve shown in Figure 3, similar to the reported “Wave of Death” (cf Figure 1 with solid curve in Figure 3).

## Discussion

Dynamic phenomena that occur during hypoxia and the way they are reflected in the EEG are only partially understood. Measurements of extreme cases showing clear features in the EEG present an opportunity to gain insight in the relation with the underlying physiology. Such an extreme case is decapitation, in which the supply of energy to the entire brain is halted almost instantaneously. This causes the EEG to become flat after several seconds, but also results in a large amplitude wave approximately a minute after decapitation. Van Rijn et al. suggest that this wave “ultimately reflects brain death” [3], but also state that further research on the physiology of brain function during this process is needed.

We modeled the membrane voltage dynamics of a single neuron with a sodium and a potassium channel and leak currents, together with the corresponding changes in the intra- and extracellular ion concentrations. This model can explain the physiological origin of the wave. When a sodium-potassium pump, glial buffering and diffusion of potassium are incorporated to model homeostasis, the model shows regular behavior and has a resting state where all variables obtain values in their physiological ranges. After shutting down the energy supply, the membrane initially depolarizes slowly with a slope of approximately 0.7 mV/s, until it reaches the excitation threshold, around −58 mV. Now spiking starts, resulting in an increase in the potassium current with a concomitant reduction in the potassium Nernst potential and membrane voltage. Positive feedback between the increasing firing rate and potassium efflux causes a sudden depolarization of the membrane voltage (30 mV in 2 seconds), resulting in the membrane depolarization curve, displayed as a dashed line in Figure 3. In combination with a high-pass filter, the simulated membrane voltage results in a wave in the EEG as observed by van Rijn et al. (Figure 3, solid line). This behavior was also observed in the in vivo measurements in rats by Siemkowicz and Hansen [5], who also measured a rapid depolarization accompanied with an increase of extracellular potassium, typically 1–2 minutes after the onset of ischemia.

While modeling the effects of decapitation, an instantaneous cessation of the sodium-potassium pump, glial buffering and diffusion of potassium to the blood was assumed. The last assumption is very reasonable, because arterial pressure vanishes after decapitation, larger vessels are drained and blood flow through the capillaries will stop. The (remaining) blood volume is relatively small and the ion concentrations in the blood will therefore quickly equilibrate with the tissue. However, a complete stop of all active ion transport will not take place directly after decapitation. Some reserves of metabolic substrates and ATP are still left in the tissue. In human brain tissue for example, these reserves can support a maximum of one minute of normal metabolism [14], but less if no oxygen is available. Such effects do not disqualify the general behavior of the model, as they will only result in a delay in the onset of depolarization, in line with the observations by van Rijn et al. Siemkowicz and Hansen [5] hypothesized that the transition from a slow to a fast rise of extracellular potassium and the corresponding depolarization is the result of depletion of these energy reserves; they hypothesized that the pumps are initially still partially fueled by anaerobic glycolysis until the glucose reserve is depleted and the ion pumps stop, causing a large efflux of potassium. We show here, however, that this is not the case and that the transition results from the Hodgkin-Huxley dynamics of the voltage dependent channels in the cell membrane.

A single neuron model was used to calculate an EEG. Although usually the network properties of neurons are essential for the EEG, we argued that a single neuron approach is realistic because synaptic transmission ceases quickly during anoxia and neurons therefore no longer receive input. Such an early cessation of transmission during hypoxia is due to failure of neurotransmitter release, presumably caused by failure of the presynaptic calcium channels [15]. Although the postsynaptic response is still intact, for example the response of the neuron to glutamate [16], neurotransmitters are no longer released and transmission is halted. The absence of significant EEG power after about 20 seconds post decapitation as observed by van Rijn et al. most likely results from this failure of synaptic transmission.

The depolarization wave was observed during a relatively short period of 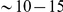 s. As the extracellular currents generated by a single pyramidal neuron are of the order of pA, much too small to generate a measurable scalp potential, a very large number of cortical neurons must simultaneously depolarize after decapitation. Such a synchronization has indeed been measured by Aitken et al., who induced depolarization in hippocampal slices, by either injecting KCl or halting the oxygen supply, to simulate spreading depression and hypoxia respectively. 1–3 minutes after the onset of hypoxia, small foci of depolarization appeared and spread with a speed of approximately 0.1 mm/s [17]. The propagation of depolarization is hypothesized to be caused by diffusion of 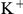 or glutamate or by interaction through gap junctions [8]. The observed speed of propagation in these slices is too slow to account for the depolarization of all cells in a whole (rat) brain in a few seconds. However, in the case of decapitation the supply of both oxygen and glucose is stopped simultaneously in the entire brain, so it is likely that many of these foci are formed simultaneously by cells of a single type with approximately the same properties. Other, fast, non-synaptic mechanisms, ephaptic transmission and electrical field effects [18], may play a role in the synchronization as well.

The absence of significant EEG power after the depolarization wave is caused by the depolarization block, which by no means implies irreversibility or cell death. Physiological cell dynamics can in principle be restored by resupplying the ion pumps with ATP. Siemkowicz and Hansen [5] observed the return of EEG activity 15 minutes after a period of isoelectricity caused by complete ischemia. High levels of intracellular calcium and cell swelling, associated with hypoxic depolarization, can be highly damaging to neurons; still this damage typically takes place over a period of hours [19]. Irreversible functional damage due to oxygen and glucose deprivation most likely occurs from damage to synapses, rather than from cell death itself [2]. In line with this perhaps surprising result, cells from neocortical slices from adult human brain obtained several hours postmortem, can survive for weeks in vitro [20]. We therefore reject the claim in the paper by van Rijn et al. [3] that this particular phenomenon can be used clinically to determine brain death. In fact, this wave does not imply death, neither of neurons nor of individuals.

In summary, our simulations and the data presented from experimental physiology show that the “Wave of Death” reflects the sudden change in membrane potential due to anoxic depolarization, that is a direct result of the Hodgkin-Huxley dynamics and ion concentrations. Although the wave is indeed a typical signature of the final membrane voltage changes of neurons suffering from severe oxygen and glucose deprivation, it is not a biomarker for irreversibility (e.g. Siemkowicz and Hansen [5]). Anoxic/hypoxic depolarizations are a well known phenomenon in experimental physiology and can be simulated with our physiologically plausible minimal model. Therefore, a more appropriate name for this phenomenon is "cerebral anoxic depolarization".

### A Parameters

Table 1 shows the parameter values used in the simulation. Values are taken from [11]. (For the correct units, refer to [11] rather than [9]). We changed the definition of 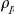 to yield a pump current density rather than a rate of change of concentration. The ratio of intra- and extracellular volume, 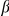, is chosen as 2 rather than 7. This is calculated from measurements by Mazel et al. that show that the extracellular space constitutes 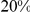 of the total tissue volume in rat brain [21] and by assuming the rest of the tissue consists of approximately the same volume of glia as neurons.

## References

[1] van Putten MJAM, van Putten MHPM (2010). Uncommon EEG burst-suppression in severe postanoxic encephalopathy.. Clin Neurophysiol.

[2] Bolay H, Grsoy-Ozdemir Y, Sara Y, Onur R, Can A (2002). Persistent defect in transmitter release and synapsin phosphorylation in cerebral cortex after transient moderate ischemic injury.. Stroke.

[3] van Rijn CM, Krijnen H, Menting-Hermeling S, Coenen AML (2011). Decapitation in rats: latency to unconsciousness and the ‘wave of death’.. PLoS One.

[4] Swaab DF, Boer K (1972). The presence of biologically labile compounds during ischemia and their relationship to the eeg in rat cerebral cortex and hypothalamus.. J Neurochem.

[5] Siemkowicz E, Hansen AJ (1981). Brain extracellular ion composition and eeg activity following 10 minutes ischemia in normo- and hyperglycemic rats.. Stroke.

[6] Dzhala V, Khalilov I, Ben-Ari Y, Khazipov R (2001). Neuronal mechanisms of the anoxia-induced network oscillations in the rat hippocampus in vitro.. J Physiol.

[7] Kager H, WadmanWJ, Somjen GG (2000). Simulated seizures and spreading depression in a neuron model incorporating interstitial space and ion concentrations.. J Neurophysiol.

[8] Somjen GG (2001). Mechanisms of spreading depression and hypoxic spreading depression-like depolarization.. Physiol Rev.

[9] Cressman JR, Ullah G, Ziburkus J, Schiff SJ, Barreto E (2009). The influence of sodium and potassium dynamics on excitability, seizures, and the stability of persistent states: I. single neuron dynamics.. J Comput Neurosci.

[10] Cressman JR, Ullah G, Ziburkus J, Schiff SJ, Barreto E (2011). Erratum to: The influence of sodium and potassium dynamics on excitability, seizures, and the stability of persistent states: I. single neuron dynamics.. J Comput Neurosci.

[11] Barreto E, Cressman JR (2011). Ion concentration dynamics as a mechanism for neuronal bursting.. Journal of Biological Physics.

[12] Hines ML, Morse T, Migliore M, Carnevale NT, Shepherd GM (2004). Modeldb: A database to support computational neuroscience.. J Comput Neurosci.

[13] Plonsey R, Barr RC (2007). Bioelectricity.. Springer, 3rd edition.

[14] Gjedde A (2007). Brain energetics: integration of molecular and cellular processes, Springer, chapter 4.

[15] Somjen GG (2004). Ions in the Brain - Normal Function, Seizures, and Stroke, Oxford University Press, chapter 18.

[16] Sun MK, Xu H, Alkon DL (2002). Pharmacological protection of synaptic function, spatial learning, and memory from transient hypoxia in rats.. J Pharmacol Exp Ther.

[17] Aitken PG, Tombaugh GC, Turner DA, Somjen GG (1998). Similar propagation of sd and hypoxic sd-like depolarization in rat hippocampus recorded optically and electrically.. J Neurophysiol.

[18] Jefferys JG (1995). Nonsynaptic modulation of neuronal activity in the brain: electric currents and extracellular ions.. Physiol Rev.

[19] Lipton P (1999). Ischemic cell death in brain neurons.. Physiol Rev.

[20] Verwer RWH, Dubelaar EJG, Hermens WTJMC, Swaab DF (2002). Tissue cultures from adult human postmortem subcortical brain areas.. J Cell Mol Med.

[21] Mazel T, Simonov Z, Sykov E (1998). Diffusion heterogeneity and anisotropy in rat hippocampus.. Neuroreport.

